# Supplementary material for: Structure-function relationship of ASH1L and histone H3K36 and H3K4 methylation
Source: Nat Commun. 2025 Mar 6;16:2235. doi: 10.1038/s41467-025-57556-5 (PMC11883000; doi:10.1038/s41467-025-57556-5)
Supplement: Supplementary file 1 — Supplementary Information [file 41467_2025_57556_MOESM1_ESM.pdf]

Supplementary Information

Structure-function relationship of ASH1L and histone H3K36 and H3K4 methylation

Kendra R. Vann, Rajal Sharma, et al.,

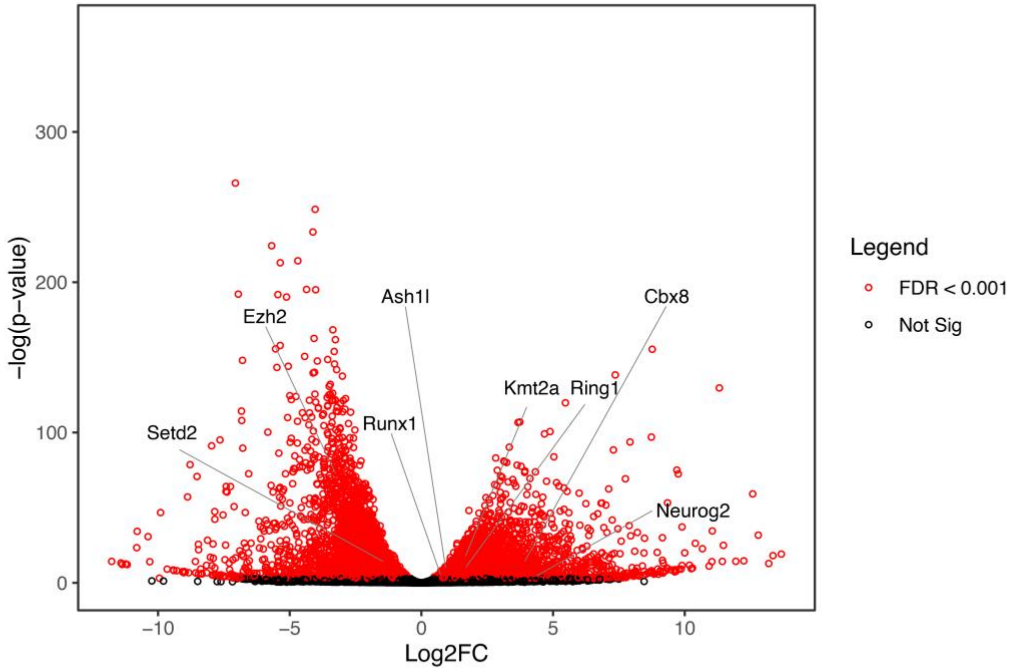

Supplementary Figure 1. Analysis of RNA-seq data reported by Yin et al. (ref. 19), comparing RNA from undifferentiated mouse ES cells to RNA from mouse ES cells differentiated under multiple conditions (see Figure 1). Gene expression changes (FC > 1.5, FDR < 0.01) in 4-day RA differentiated cells were identified using DESeq2, with undifferentiated cells as controls. A Volcano plot highlights significantly upregulated genes after RA treatment. Related to Figure 1.

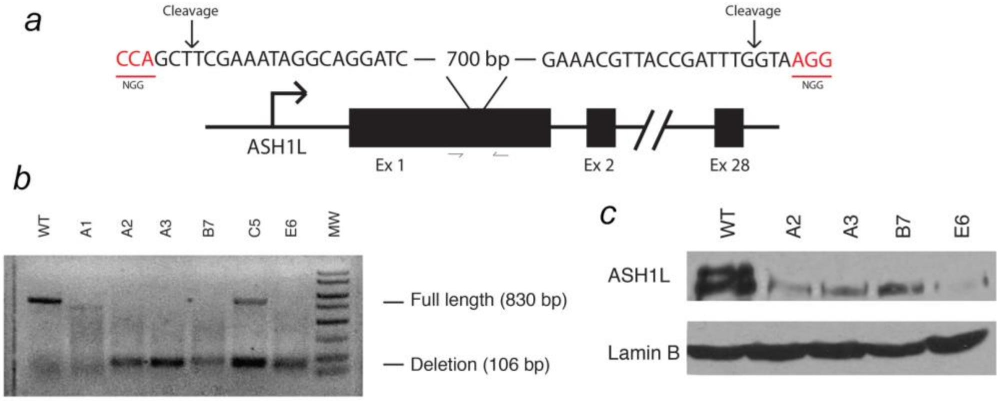

Supplementary Figure 2. Design of Ash1L knockout in mouse ES cells by CRISPR/Cas9. (a) A scheme depicting CRISPR/Cas9 mediated editing of Ash1l in mouse ES cells. Two guide RNAs were designed to flank nearly 750 bps of exon 1. Also annotated are the two PCR primers used to check clones for success of the gene editing. (b) PCR analysis of multiple clones isolated from CRISPR/Cas9 editing. PCR primers labeled in panel (a) were used to amplify a region of exon 1 targeted by gRNAs. The full-length product and the edited product are illustrated. (c) Western blot analysis showing Ash1l protein expression levels in edited clones after two days of retinoic acid mediated differentiation of mouse ES cells. Antibodies used: Ash1l (Bethyl, A301-749A; 1:1000), Lamin B (Santa Cruz, sc-373918; 1:3000). Related to Figure 1. Source data are provided with this paper.

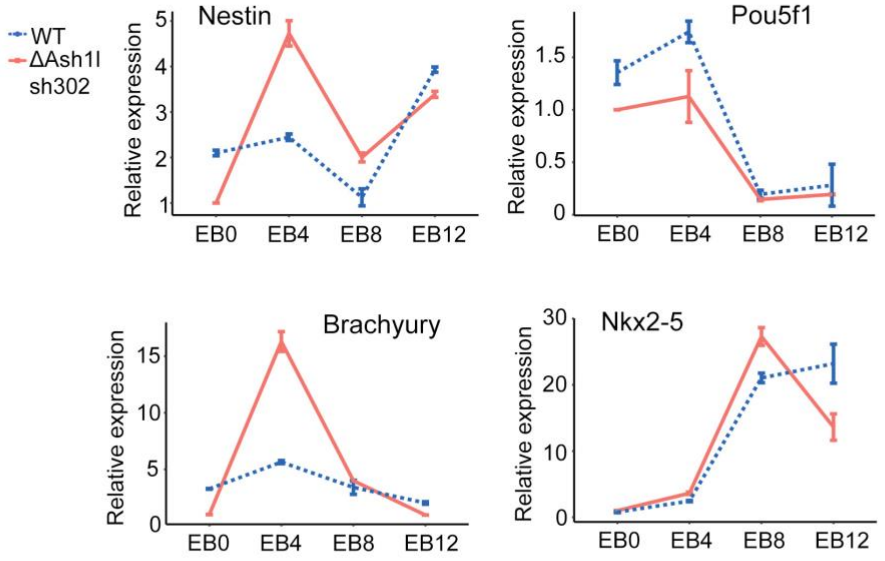

Supplementary Figure 3. Effect of the Ash1L deletion on gene expression. Relative expression of key differentiation genes during embryoid body development in WT or ∆Ash1l mES cells transfected with shLuc or shAsh1l. Related to Figure 1.

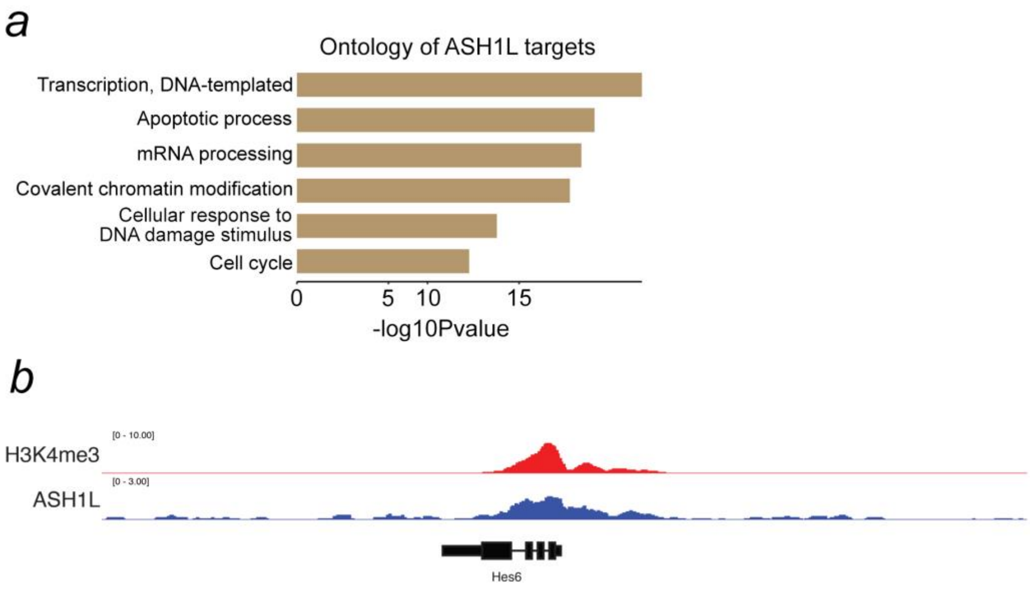

Supplementary Figure 4. Genomic distribution of Ash1l. (a) Gene ontology (GO) analysis of direct Ash1l target genes identified by ChIP-seq in RA differentiated mES cells. (b) Genome browser snapshot of Ash1l and H3K4me3 occupancy at the *Hes6* locus. Related to Figure 2.

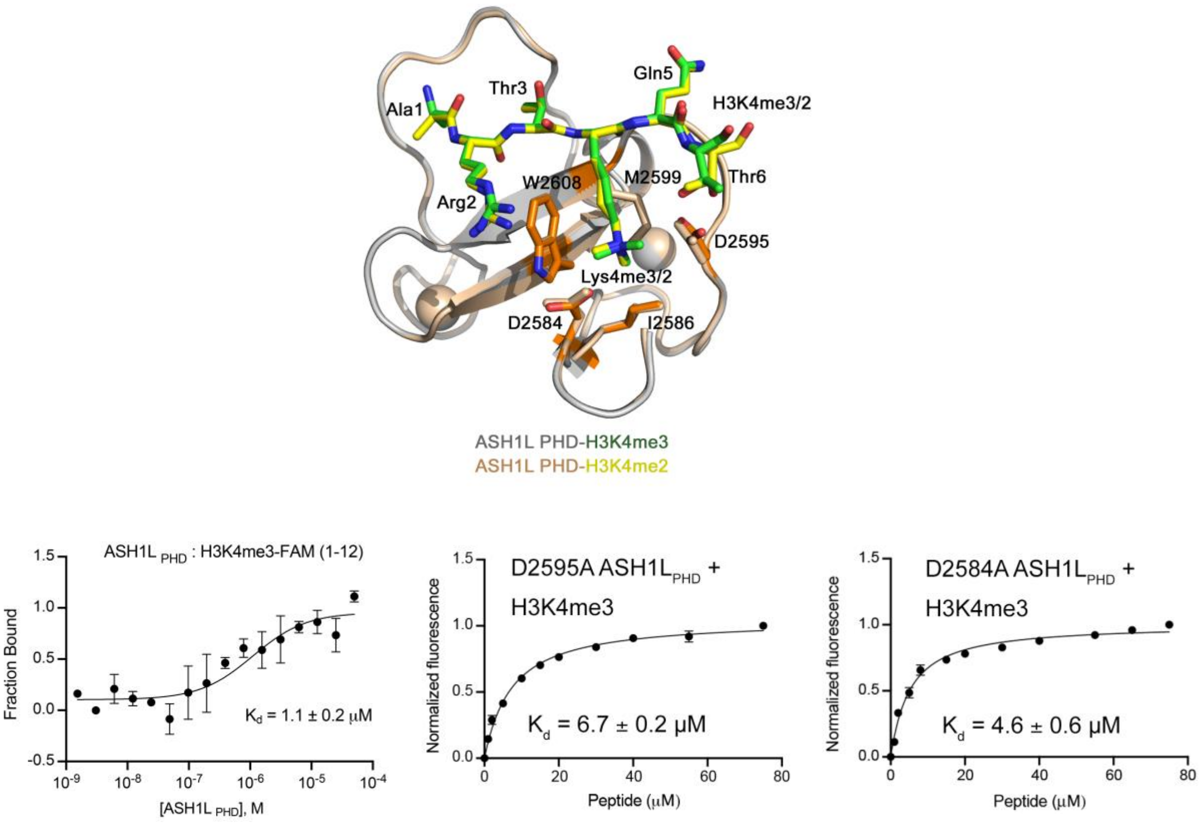

Supplementary Figure 5. (top) Comparison of the $ASH1L_{PHD}$ complexes. Overlay of the crystal structures of $ASH1L_{PHD}$ in complex with H3K4me3 (green) and H3K4me2 (yellow). The aromatic cage and surrounding residues are depicted as sticks and labeled. (below, left) Binding affinity of $ASH1L_{PHD}$ to H3K4me3-FAM (aa 1-12 of H3) peptide was measured by MST. The K_d value represents the average of four independent measurements ± SEM. Point errors represent SEM. (below, middle and right) Binding curves used to determine K_d values of indicated mutants of $ASH1L_{PHD}$ to H3K4me3 by tryptophan fluorescence. K_ds are calculated as mean values +/- S.D. from three independent experiments. Related to Figure 3.

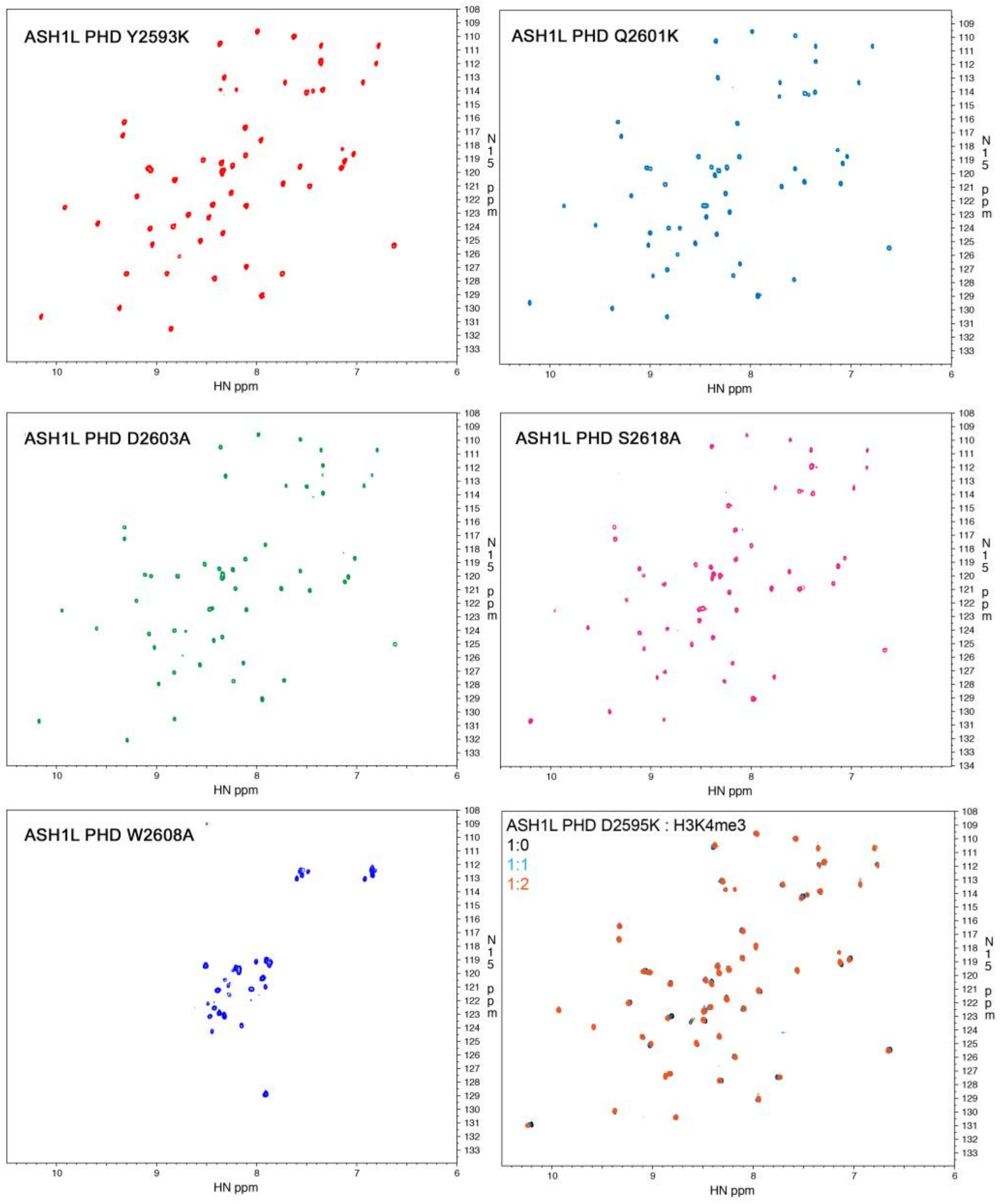

Supplementary Figure 6. ^{1}H,^{15}N HSQC spectra of the indicated mutants of ASH1L$_{PHD}$. (bottom right) Overlay of ^{1}H,^{15}N HSQC spectra of D2595K ASH1L$_{PHD}$ in the absence or presence of H3K4me3 peptide. Spectra are color coded according to the protein:peptide molar ratio. Related to Figure 3.

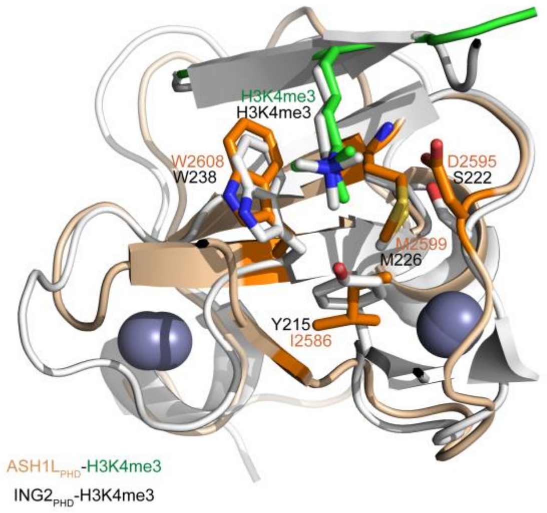

Supplementary Figure 7. Overlay of the crystal structures of ASH1L$_{PHD}$ (wheat) in complex with H3K4me3 (green) and ING2$_{PHD}$ (grey) in complex with H3K4me3 (grey) (PDB 1G6Q). Aromatic cage residues are depicted as sticks and labeled. Related to Figure 3.

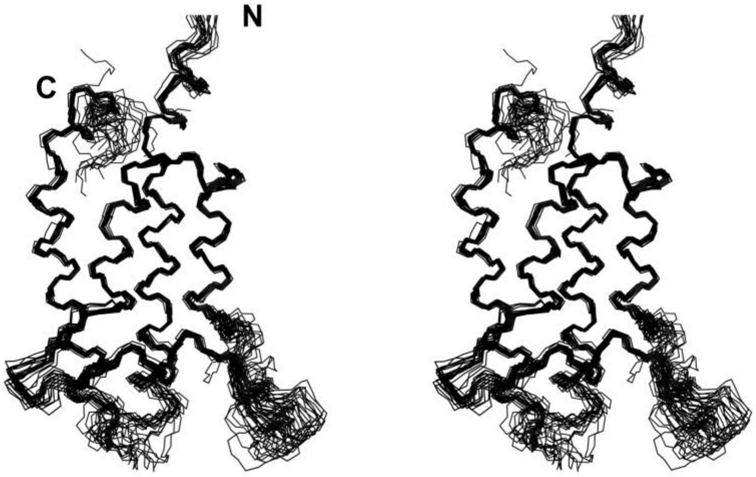

BD

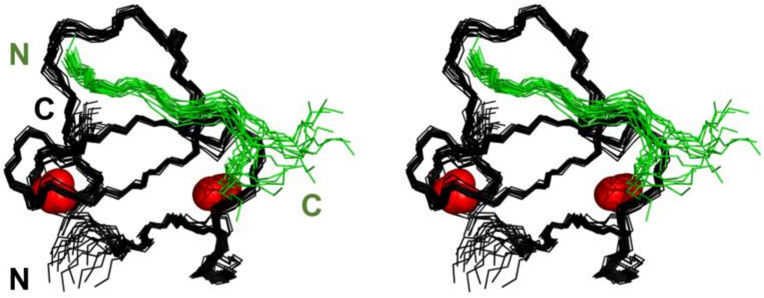

PHD-H3K4me2

Supplementary Figure 8. NMR structures ASH1L$_{BD-PHD}$ in complex with H3K4me2 peptide. Superimposition of the final 20 NMR structures of ASH1L$_{BD}$ (upper) and ASH1L$_{PHD}$ bound to H3K4me2 peptide (lower). The structurally disordered loop connecting the BD and PHD is omitted for clarity. The H3K4me2 peptide and two zinc atoms are colored in green and red, respectively. Related to Figure 4.

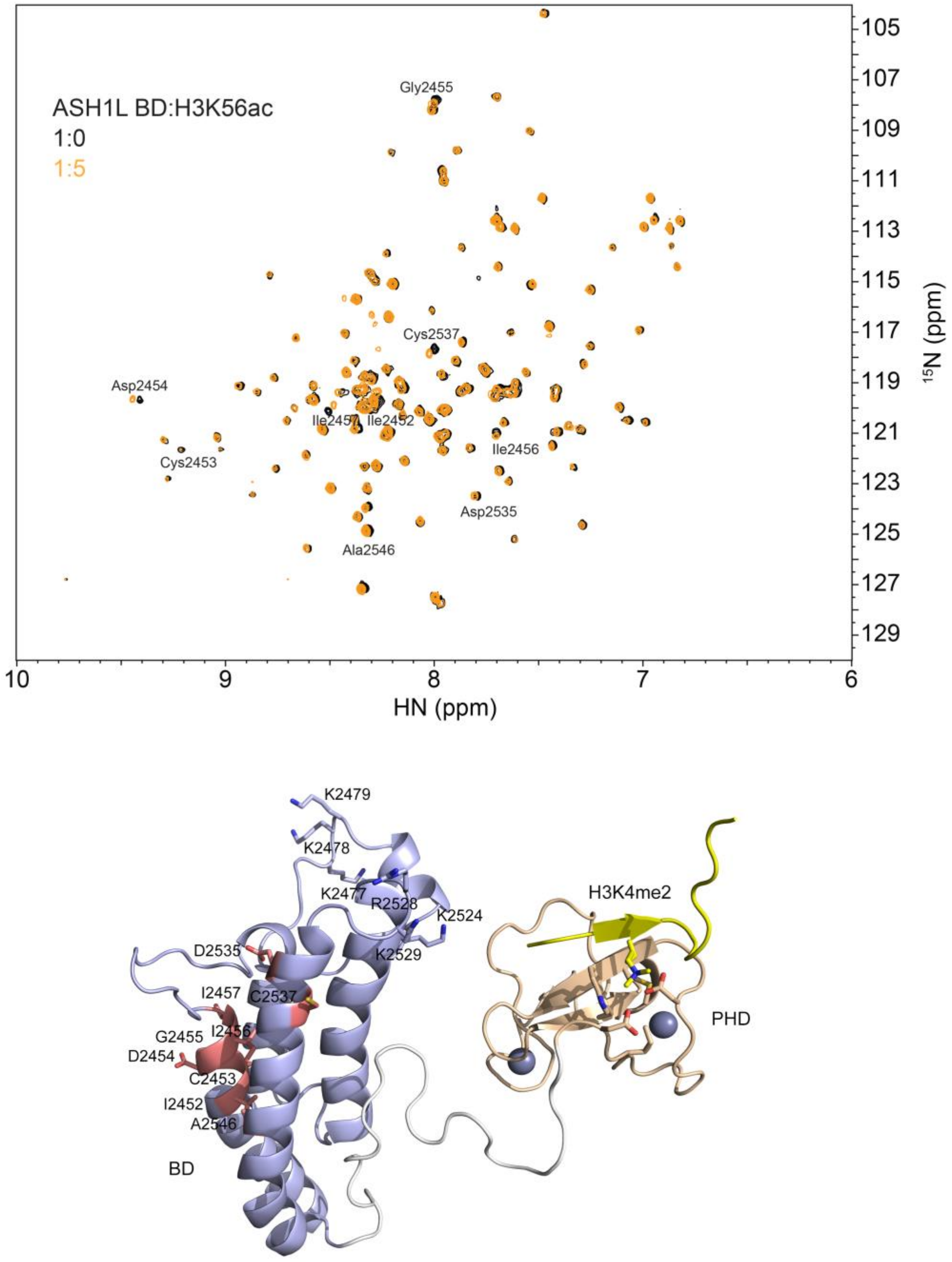

Supplementary Figure 9. Mapping the H3K56ac binding site of $ASH1L_{BD}$. (top) Superimposed ^{1}H,^{15}N HSQC spectra of $ASH1L_{BD}$ in the absence (black) or presence (orange) of 5 molar excess of H3K56ac peptide. Residues of $ASH1L_{BD}$ that exhibit chemical shift perturbations are colored salmon and mapped onto the crystal structure of $ASH1L_{BD\text{-}PHD}$ in complex with H3K4me2 (bottom). Residues of $ASH1L_{BD}$ involved in binding to DNA are shown as blue sticks and labeled. Related to Figure 4.

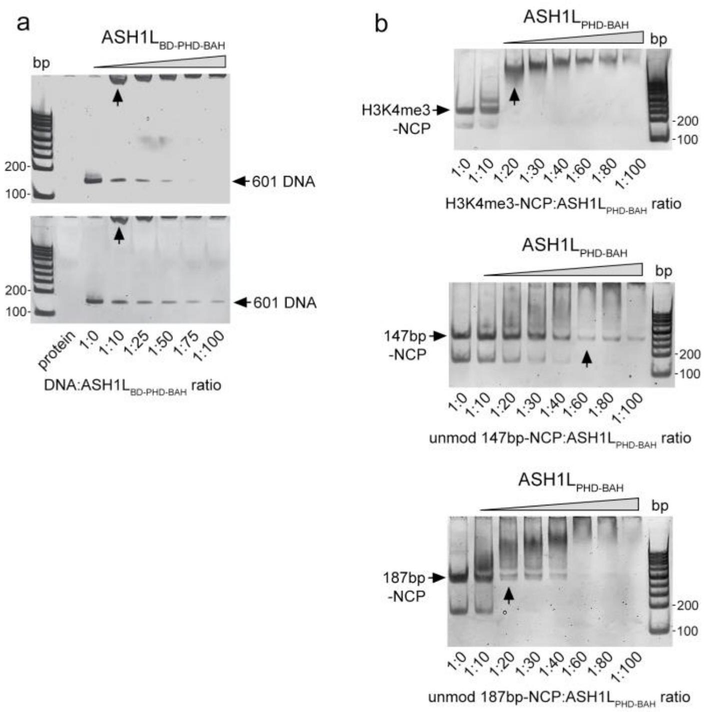

Supplementary Figure 10. (a) Duplicate of EMSA with 147 bp 601 DNA in the presence of increasing amounts of ASH1L$_{BD-PHD-BAH}$. Arrows indicate the DNA-ASH1L$_{BD-PHD-BAH}$ complex. (b) EMSAs of 147 bp H3K4me3-NCP (top, also shown in Fig. 4h), unmodified 147 bp NCP (middle) and unmodified 187 bp NCP (bottom) in the presence of increasing amounts of ASH1L$_{PHD-BAH}$. Lanes where complexes are predominantly formed are indicated by arrows. Related to Figure 5.

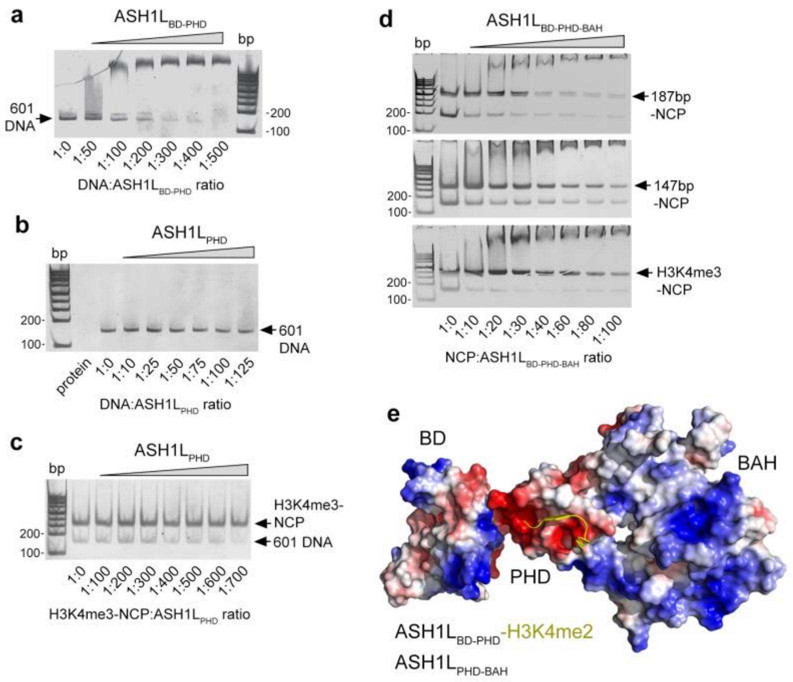

Supplementary Figure 11. (a) EMSA of 147 bp 601 DNA in the presence of increasing amounts of ASH1L$_{BD-PHD}$. DNA binding of ASH1L$_{BD-PHD}$ is decreased compared to the binding of isolated ASH1L$_{BD}$ likely due to the negatively charged linker that connects two domains as well as the negatively charged surface of PHD, however the tight binding to DNA is restored for ASH1L$_{BD-PHD-BAH}$. (b, c) EMSA of 147 bp 601 DNA or H3K4me3-NCP in the presence of increasing amounts of ASH1L$_{PHD}$ shows that isolated ASH1L$_{PHD}$ is incapable of binding to H3K4me3-NCP, corroborating previous studies reporting that histone tails, being bound to the nucleosomal DNA, are not easily accessible and have to be released. (d) EMSA of 187bp-NCP, H3K4me3-NCP, and 147bp-NCP in the presence of increasing amounts of ASH1L$_{BD-PHD-BAH}$. The preference for 187bp-NCP and H3K4me3-NCP over 147bp-NCP is maintained in ASH1L$_{BD-PHD-BAH}$. (e) Overlay of the structures of H3K4me2-bound ASH1L$_{BD-PHD}$ and ASH1L$_{PHD-BAH}$ as in Figure 6k, but shown as surfaces. Electrostatic surface potential ranging from positive;blue (+100 kT/e) to negative;red (−100 kT/e) was generated with PyMol vacuum electrostatics. Related to Figure 5.

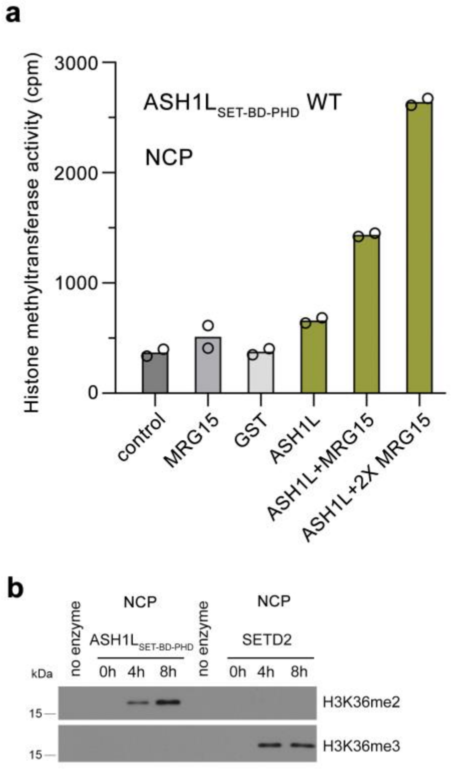

Supplementary Figure 12. (a) Lysine methyltransferase activity of recombinant ASH1L$_{SET-BD-PHD}$ in the absence and presence of MRG15 on native chromatin substrate from HeLa cells. Experiment was performed twice. (b) Western blot analysis of *in vitro* KMT assays of ASH1L on a recombinant nucleosome. Blots were probed with H3K36me2 and H3K36me3 antibodies. SETD2 was used for comparison. Related to Figure 6.

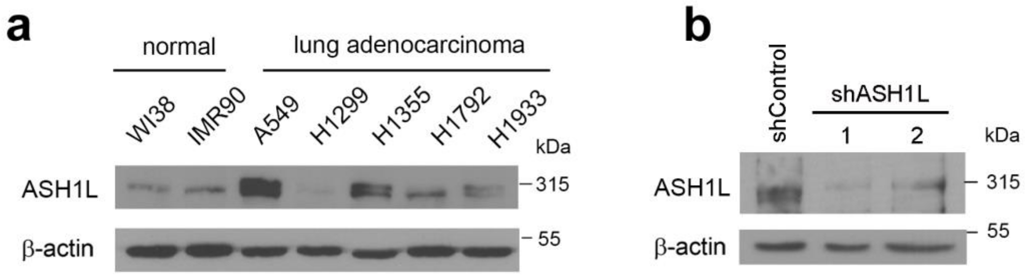

Supplementary Figure 13. ASH1L is amplified in lung adenocarcinoma cell lines. (a) Western blot analysis of ASH1L protein expression levels in the indicated lung adenocarcinoma cell lines and control normal cells. Actin is used as a loading control. (b) Western blot analysis of ASH1L knockdown in A549 cells. Related to Figure 6.

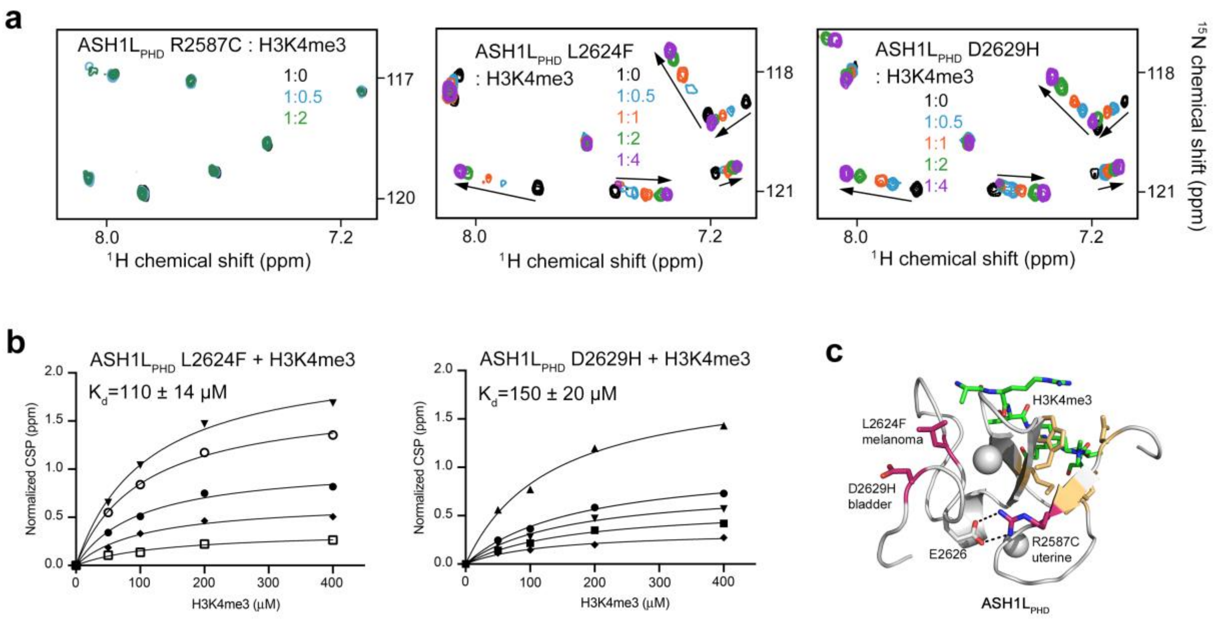

Supplementary Figure 14. A number of mutations associated with cancer have been identified in ASH1L$_{PHD}$ (Cosmic). (a) Superimposed ^{1}H,^{15}N HSQC spectra of cancer-relevant mutants of ASH1L$_{PHD}$ recorded in the presence of increasing amounts of the H3K4me3 peptide. The spectra are color coded according to the protein:peptide molar ratio. (b) Binding curves used to determine K_d values by NMR. K_ds are represented as mean values +/- S.D. (c) The structure of ASH1L$_{PHD}$ in complex with H3K4me3 (green) with mutated residues shown as magenta sticks and labeled. Related to Figure 6.

Supplementary Table 1. X-ray diffraction data collection and refinement statistics

	$ASH1L_{PHD-BAH}$	$ASH1L_{PHD}$-H3K4me3	$ASH1L_{PHD}$-H3K4me2
**Data Collection**			
Space group	C 1 2 1	P 43	P 43
Wavelength (Å)	1.54	1.28	1.28
Resolution (Å)	50.0- 2.4 (2.47-2.40)*	20.0-1.3 (1.39-1.34)*	35.3-1.9 (1.99-1.92)*
Unit cell lengths a, b, c	173.1 60.6 53.6	49.80 49.80 46.29	49.96 49.96 46.05
Unite cell angles α, β, γ	90.0 97.2 90.0	90.0 90.0 90.0	90.0 90.0 90.0
No. of measured reflections	59159	142736	158174
No. of unique reflections	20953	25506	8755
Multiplicity	2.8 (1.6)	5.6 (4.6)	18.0 (10.0)
I/σ	12.2 (2.3)	12.5 (3.2)	31.0 (12.3)
Completeness (%)	97.0 (88.2)	100 (99.8)	99.9(99.3)
R_{merge}(%)	6.3 (31.9)	5.2 (41.3)	6.1(9.6)
No. of molecules in ASU	2	2	2
Solvent content (%)	50.0	48.9	49.0
**Refinement**			
R_{work}	23.0 (29.7)	15.49 (22.61)	20.1 (26.5)
R_{free}	28.4 (32.3)	18.83 (25.14)	24.9 (31.7)
No. of atoms	4259	1141	1033
Protein	4109	1014	936
Water	145	123	93
B-factors ($Å^2$)	43.1	29.61	29.27
R.M.S.D			
Bond lengths (Å)	0.002	0.006	0.005
Bond angles (°)	0.74	0.96	0.92
Ramachandran favored (%)	96	96	97
Ramachandran allowed (%)	4	4	3
Ramachandran outliers	0.2	0	0
Clashscore	4	1.0	3.8

*Values in parentheses are for the highest resolution shell (Å).

#$R_{merge} = \sum|I_{obs}-I_{calc}|/I_{obs}$, where I_{obs} is intensity of any given reflection and I_{avg} is the weighted mean I.

Supplementary Table 2. Summary of restraints and statistics of the final 20 out of 200 structures of the ASH1L$_{BD-PHD}$

Protein NMR distance and dihedral constraints	
Distance constraints	
Total NOE	2645
Intra-residue	1027
Inter-residue	1618
Sequential (|i - j| = 1)	325
Medium-range (1 < |i - j| ≤ 5)	703
Long-range (|i - j| > 5)	590
Inter-molecular constraints	25
Hydrogen bonds	83
Total dihedral angle restraints	
Phi angle	141
Psi angle	141
Ramachandran Map Analysis (%)[a]	
Most favored regions	95.0
Additional allowed regions	5.0
Generally allowed regions	0.0
Disallowed regions	0.0
Structure statistics[c]	
Violations (mean +/- s.d.)	
Distance constraints (Å)	0.13 +/- 0.11
Dihedral angle constraints (°)	0.70 +/- 0.49
Max. dihedral angle violation (°)	1.7
Max. distance constraint violation (Å)	0.35
Deviations from idealized geometry	
Bond lengths (Å)	0.0049+/- 0.00023
Bond angles (°)	0.65 +/- 0.031
Improper (°)	1.7 +/- 0.14
Average pairwise r.m.s. Deviation (Å)[b]	
Heavy	0.53 +/- 0.097 (BD domain)
Backbone	0.46 +/- 0.085 (BD domain)
Heavy	0.56 +/- 0.11 (PHD domain)
Backbone	0.48 +/- 0.15 (PHD domain)

[a]: Procheck calculation was done for protein residues 2438-2459, 2493-2503, 2508-2527, 2531-2556, 2585-2586, 2597-2603, 2605-2617, 2623-2628.

[b]: The residue number ranges used in full molecule pairwise root-mean-square (r.m.s.) deviation calculations consist of 2438-2459, 2465-2474, 2493-2507, 2508-2527, 2531-2557, 2585-2629 for PHD.

[c]: Pairwise r.m.s. deviation was calculated among top 20/200 lowest energy structures.
