# Supplementary material for: Structure-function relationship of ASH1L and histone H3K36 and H3K4 methylation
Source: Nat Commun. 2025 Mar 6;16:2235. doi: 10.1038/s41467-025-57556-5 (PMC11883000; doi:10.1038/s41467-025-57556-5)
Supplement: Supplementary file 2 — Description of Additional Supplementary Files [file 41467_2025_57556_MOESM2_ESM.pdf]

Description of Additional Supplementary Files

Supplementary Data 1 - RNA-seq analysis of shASH1L treated A549 cells.
