# Supplementary material for: Structure-function relationship of ASH1L and histone H3K36 and H3K4 methylation
Source: Nat Commun. 2025 Mar 6;16:2235. doi: 10.1038/s41467-025-57556-5 (PMC11883000; doi:10.1038/s41467-025-57556-5)
Supplement: Supplementary file 4 — Reporting Summary [file 41467_2025_57556_MOESM4_ESM.pdf]

nature portfolio

Corresponding author(s): Tatiana Kutateladze
Ming-Ming Zhou

Last updated by author(s): Feb 12, 2025

Reporting Summary

Nature Portfolio wishes to improve the reproducibility of the work that we publish. This form provides structure for consistency and transparency in reporting. For further information on Nature Portfolio policies, see our Editorial Policies and the Editorial Policy Checklist.

Statistics

For all statistical analyses, confirm that the following items are present in the figure legend, table legend, main text, or Methods section.

n/a	Confirmed	
☐	☒	The exact sample size (*n*) for each experimental group/condition, given as a discrete number and unit of measurement
☐	☒	A statement on whether measurements were taken from distinct samples or whether the same sample was measured repeatedly
☐	☒	The statistical test(s) used AND whether they are one- or two-sided<br>*Only common tests should be described solely by name; describe more complex techniques in the Methods section.*
☒	☐	A description of all covariates tested
☐	☒	A description of any assumptions or corrections, such as tests of normality and adjustment for multiple comparisons
☐	☒	A full description of the statistical parameters including central tendency (e.g. means) or other basic estimates (e.g. regression coefficient) AND variation (e.g. standard deviation) or associated estimates of uncertainty (e.g. confidence intervals)
☐	☒	For null hypothesis testing, the test statistic (e.g. *F*, *t*, *r*) with confidence intervals, effect sizes, degrees of freedom and *P* value noted<br>*Give P values as exact values whenever suitable.*
☒	☐	For Bayesian analysis, information on the choice of priors and Markov chain Monte Carlo settings
☒	☐	For hierarchical and complex designs, identification of the appropriate level for tests and full reporting of outcomes
☐	☒	Estimates of effect sizes (e.g. Cohen's *d*, Pearson's *r*), indicating how they were calculated

Our web collection on statistics for biologists contains articles on many of the points above.

Software and code

Policy information about availability of computer code

Data collection: RT-qPCR and ChIP-qPCR data were collected with the Mx3005P Real-Time PCR System (Agilent). Sequencing was performed on the Illumina NovaSeq platform. NMR experiments were performed on Varian 600 MHz and 900 MHz spectrometers and Bruker 900 and 800 MHz NMR spectrometers. Tryptophan fluorescence spectra were recorded on a Fluoromax-3 spectrofluorometer (HORIBA). Microscale thermophoresis (MST) experiments were performed using a Monolith NT.115 instrument (NanoTemper). X-ray diffraction data were collected at the National Synchrotron Light Source using beamline X25 and on a Rigaku Micromax 007 high-frequency microfocus X-ray generator in CU Anschutz X-ray crystallography core facility.

Data analysis

Statistics:
PRISM (GraphPad v8)

ChIP-Seq:
Trimmomatic v0.36
Bowtie v1.0.1
Samtools v1.2
MACS2 v2.1.1
deepTools v2.0
IGV Genome Browser (v2.17.0).
Software for structure determination include iMOSFLM, SCALA, XDS, Phenix_Refine, COOT, MrBUMP, BUCANEER, MolProbity and REFMAC, as well as NMRPipe, NMRVIEW, CNS, ARIA and PROCHECK as listed in method section. Fluorescence data were analyzed using GraphPad Prism v8. MST software for calculation of Kd include MO Affinity Analysis as listed in method section.

For manuscripts utilizing custom algorithms or software that are central to the research but not yet described in published literature, software must be made available to editors and reviewers. We strongly encourage code deposition in a community repository (e.g. GitHub). See the Nature Portfolio guidelines for submitting code & software for further information.

Data

Policy information about availability of data

All manuscripts must include a data availability statement. This statement should provide the following information, where applicable:

- Accession codes, unique identifiers, or web links for publicly available datasets
- A description of any restrictions on data availability
- For clinical datasets or third party data, please ensure that the statement adheres to our policy

Coordinates and structure factors have been deposited in the Protein Data Bank under the accession numbers 8VLD [https://doi.org/10.2210/pdb8VLD/pdb], 8VLF [https://doi.org/10.2210/pdb8VLF/pdb], 8VLH [https://doi.org/10.2210/pdb8VLH/pdb], and 8ZXC [https://doi.org/10.2210/pdb8ZXC/pdb]. NMR data have been deposited in the Biological Magnetic Resonance Bank under accession number 36675 [https://doi.org/10.13018/BMR36675]. The ChIP-seq data are deposited to GEO: https://www.ncbi.nlm.nih.gov/geo/query/acc.cgi?acc=GSE199438. The RNA-seq data are deposited to GEO: https://www.ncbi.nlm.nih.gov/geo/query/acc.cgi?&acc=GSE198706. All other relevant data supporting the key findings of this study are available within the article and Supplementary Information files. Source data are provided with this paper.

Research involving human participants, their data, or biological material

Policy information about studies with human participants or human data. See also policy information about sex, gender (identity/presentation), and sexual orientation and race, ethnicity and racism.

Reporting on sex and gender	N/A
Reporting on race, ethnicity, or other socially relevant groupings	N/A
Population characteristics	N/A
Recruitment	N/A
Ethics oversight	N/A

Note that full information on the approval of the study protocol must also be provided in the manuscript.

Field-specific reporting

Please select the one below that is the best fit for your research. If you are not sure, read the appropriate sections before making your selection.

☒ Life sciences ☐ Behavioural & social sciences ☐ Ecological, evolutionary & environmental sciences

For a reference copy of the document with all sections, see nature.com/documents/nr-reporting-summary-flat.pdf

Life sciences study design

All studies must disclose on these points even when the disclosure is negative.

Sample size	The chosen sample sizes are based on our experience with each technique and as reported in prior publications. They are present in the relevant figure legends.
Data exclusions	No samples were excluded

Replication	As noted in the relevant figure legends.
Randomization	Randomization was not relevant to our presented studies.
Blinding	Blinding was not relevant to our presented studies.

Reporting for specific materials, systems and methods

We require information from authors about some types of materials, experimental systems and methods used in many studies. Here, indicate whether each material, system or method listed is relevant to your study. If you are not sure if a list item applies to your research, read the appropriate section before selecting a response.

Materials & experimental systems

n/a	Involved in the study
☐	☒ Antibodies
☐	☒ Eukaryotic cell lines
☒	☐ Palaeontology and archaeology
☒	☐ Animals and other organisms
☒	☐ Clinical data
☒	☐ Dual use research of concern
☒	☐ Plants

Methods

n/a	Involved in the study
☐	☒ ChIP-seq
☒	☐ Flow cytometry
☒	☐ MRI-based neuroimaging

Antibodies

Antibodies used	Antibodies used for ChIP-seq assays and WB in mESCs: Ash1l Bethyl A301-749A (ChIP: 5 µg/20 million cells, WB: 1:1000), Lamin B Santa Cruz sc-373918 (WB: 1:3000), H3K36me2 Abcam ab9049 (ChIP: 3 µg/3 million cells), H3K4me3 Abcam ab8580 (ChIP: 3 µg/3 million cells).<br>Antibodies used in KMT assays: GST (Sata Cruz, Sc-459; 1:1000), H4 (Abcam, ab7311; 1:500), H3K4me3 (Abcam, ab8580; 1:1000), H3K36me1 (Abcam, ab9048; 1:1000), H3K36me2 (Abcam, ab9049; 1:5000), H3K36me3 (Abcam, ab9050; 1:5000).
Validation	Histone antibodies were validated as indicated on their manufacturer's website and by prior publications. ASH1L antibody was validated in this manuscript by shRNA knockdown and CRISPR knockout followed by western blotting. All antibodies used for western blotting showed bands corresponding to the expected molecular weight.

Eukaryotic cell lines

Policy information about cell lines and Sex and Gender in Research

Cell line source(s)	HEK293T cells, used for viral packaging, were obtained from Takara Bio, Cat. #632180. J1 mouse embryonic stem cells were a kind gift from Jianlong Wang, PhD.
Authentication	HEK293T cells were authenticated by the manufacturer. J1 mouse embryonic stem cells were authenticated by RNA-seq in prior publications (PMID:35353581).
Mycoplasma contamination	All cell lines were routinely tested for Mycoplasma using the LookOut Mycoplasma PCR Detection Kit (Sigma-Aldrich).
Commonly misidentified lines<br>(See ICLAC register)	No commonly misidentified cell lines were used in this study.

Plants

Seed stocks	N/A
Novel plant genotypes	N/A
Authentication	N/A

ChIP-seq

Data deposition

☒ Confirm that both raw and final processed data have been deposited in a public database such as GEO.

☒ Confirm that you have deposited or provided access to graph files (e.g. BED files) for the called peaks.

Data access links<br>*May remain private before publication.*	ChIP-seq data sets were deposited to GEO, under GSE199438 (https://www.ncbi.nlm.nih.gov/geo/query/acc.cgi?acc=GSE199438).
Files in database submission	GSM5973022 Ash1l_mES<br>GSM5973023 Input_mES
Genome browser session<br>(e.g. UCSC)	https://genome.ucsc.edu/s/rsharma128/ASH1L

Methodology

Replicates	For each condition, one IP was generated after validation with multiple ChIP-qPCR experiments. This approach was in agreement with prior studies (PMID:37605008).
Sequencing depth	Sample Number Mapped Reads Total reads Read Length Single vs Paired<br>WT-ASH-1 37958058 49254540 50bp Single-end<br>WT-Input 31226319 35358664 50bp Single-end
Antibodies	Ash1l (Bethyl, A301-749A; 5 µg), H3K36me2 (Abcam, ab9049; 3 µg), H3K4me3 (Abcam, ab8580; 3 µg)
Peak calling parameters	Mapping:<br>gzip -dc $(gz file) | bowtie -p 8 -S $(mm9 genome) - > $(sam file)<br>samtools view -bSh -F 4 $(sam file) | samtools sort - $(sorted sam)<br><br>Peak calling:<br>macs2 callpeak -t $(treated bam file) -c $(input bam file) -f BAM -g mm -n $(out file name) -q .05 --keep-dup=auto --nomodel --extsize=150
Data quality	Peaks Above 5FE Peaks Below 5FE %Above 5FE<br>WT-ASH1L-1 12185 9639 79.1%
Software	After quality control and read filtering using Trimmomatic v0.36, reads were aligned to the mouse reference genome (mm9) using Bowtie (v1.0.1). Peaks were identified from aligned reads using MACS2 (v2.1.1) with FDR < 0.05.
